# Fungal keratitis caused by *Pseudallescheria boydii*: clinical and mycological characteristics

**DOI:** 10.1186/s12348-021-00255-1

**Published:** 2021-09-24

**Authors:** Alireza Izadi, Mohammad Soleimani, Claudy Oliveira dos Santos, Marlou C. Tehupeiory-Kooreman, Roshanak Daie Ghazvini, Seyed Jamal Hashemi, Mohssen Gramishoar, Mehdi Aminizadeh, Zohre Abedinifar, Paul E. Verweij, Sadegh Khodavaisy

**Affiliations:** 1 Department of Medical Parasitology and Mycology, School of Public Health, Tehran University of Medical Sciences, Tehran, Iran; 2 Department of Ocular Trauma and Emergency, Farabi Eye Hospital, Tehran University of Medical Sciences, Tehran, Iran; 3 Department of Medical Microbiology and Center of Expertise in Mycology Radboudumc/CWZ, Radboud University Medical Centre, Nijmegen, The Netherlands; 4 Eye Research Center, Farabi Eye Hospital, Tehran University of Medical Sciences, Tehran, Iran

**Keywords:** Fungal keratitis, *Pseudallescheria boydii*, Antifungal

## Abstract

**Background:**

Pseudallescheria keratitis is rare but important type of fungal keratitis because of the inherently resistance of the organism to many existing antifungal agents.

**Methods:**

Slit-lamp and confocal microscopy were used for clinical examinations. Fungal isolates were identified based on morphological characteristics and DNA sequence of the internal transcribed spacer region (ITS). In vitro antifungal susceptibility testing for fungal isolates was performed according to the Clinical and Laboratory Standards Institute (CLSI, M38-A2).

**Result:**

All patients had a history of ocular trauma. In clinical examination hypopion were seen in three patients. The main antifungal medications were topical voriconazole. After treatment the visual acuity of all patients improved in 2–3 weeks.

**Conclusion:**

All four patients of Pseudallescheria keratitis had similar clinical features. Accurate and rapid identification of species should be helpful in treating *p. boydii* keratitis.

## Introduction

Fungal keratitis is not life threatening but certainly cause ocular morbidity and sight threatening all over the world [1]. It has been caused by fungal species that capable to colonize and invade the ocular surface and corneal stroma [2]. The etiologic and epidemiologic pattern of fungal keratitis varies depending on patient status, geographic location, and climate [3]. Fungal keratitis can cause by at least 35 genera of moulds and yeasts [3, 4]. *Pseudallescheria boydii* (previously *Petriellidium boydii*, *Allescheria boydii*, *Monosporium apiospermum*) belongs to the group of septate filamentous fungi, which are the most common pathogenic fungi causing opportunistic human infections associated with trauma [5, 6]. This saprophytic fungus frequently isolated from soil, polluted water, sewage, decaying organic substances, manure, and potted plants throughout the world [7]. *P. boydii* implicated in a wide range of infections (mycetoma, pneumonia, osteomyelitis, arthritis, sinusitis, endocarditis, meningitis, and brain abscess) in both immune-compromised and immune-competent patients [7]. Several reports have documented eye infections such as keratitis, scleritis, sclerokeratitis, chorioretinitis, and endophthalmitis due to *P. boydii* following injury, herpetic keratitis and operation [8]. The first keratitis caused by *Scedosporium* species reported in 1955 [9]. The prognosis of fungal keratitis caused by *P. boydii* is poor [7]. Treatment of these pathologies is difficult because of the inherently resistance of the organism to many existing antifungal agents [8, 9]. We present the clinical characteristics, risk factors, treatment, and prognosis of four patients with *P. boydii* keratitis, and also present the antifungal sensitivities of the isolated strain.

## Methods

### Subjects

Slit-lamp and in vivo confocal microscopy (IVCM) (Heidelberg Engineering, Heidelberg, Germany) were used for clinical examinations. The clinical characteristics of four patients with Pseudallescheria keratitis were reviewed.

### Sample collection and processing

Corneal scraping material was collected after anaesthetising the cornea with instillation of drops of 0.5% proparacaine and cleaning the cornea with sterile normal saline to remove all necrotic exudates. After 5 min with the help of blade no. 15 scraping was done under slit lamp illumination by the ophthalmologist. Corneal scrapings were sent to Medical Mycology laboratory, Tehran University of Medical Sciences, for mycology studies. Gram staining, 10% Potassium Hydroxide (KOH) mount, culture on sabouraud dextrose agar with chloramphenicol and slide culture was carried out to identify the isolate up to the genus level.

### Molecular identification

One colony of each fungal species scraped and DNA was extracted using a DNA isolation kit (Gene All DNA extraction kit; Gene All, Germany) according to the manufacturer’s instructions. To confirm the *Pseudallescheria* species identification, the Internal Transcribed Spacer (ITS) region was sequenced [10]. ITS 1 (5′-CGC TGC GTT CTT CAT CG-3′) and ITS 2 (5′-TCG ATG AAG AAC GCA GCG-3′) primers were used for amplification. Yielded sequence was subjected to Basic Local Alignment Search Tool (BLAST) program (http://www.blast.ncbi.nlm.nih.gov/Blast).

### Antifungal susceptibility

In vitro antifungal susceptibility testing was performed according to the Clinical and Laboratory Standards Institute (CLSI) M38-A2 for filamentous fungi [11]. *Pseudallescheria* species susceptibility of amphotricin B, 5 flucytosine, fluconazole, itraconazole, voriconazole, posaconazole, miconazole, caspofungin, chlorhexidin and natamycin was tested by determining minimum inhibitory concentration (MICs).

## Results

### Clinical characteristics

Data regarding age, sex, occupation, detailed clinical history, clinical presentation and symptoms are summarized in Table 1.

**Table 1:** Clinical details of patients involved in this study

Patient No.	Sex	Age	Job	Risk factor	Side	Size	VA1	VA2	Treatment	Hypopion	PKP
**1**	Female	46	House wife	Injured by dust particle	OD	3 mm	CF	20/160	VRC, ITR, AMB	Yes	No
**2**	Male	69	Farmer	Trauma with plant material	OS	4 mm	HM	20/200	VRC	Yes	No
**3**	Male	30	Woodworker	Injured by wood particle	OS	3.5 mm	CF	20/19	VRC	Yes	No
**4**	Male	37	Farmer	Trauma with plant material	OD	4 mm	20/60	20/16	VRC	No	No

Abbreviations: *OS* Oculus sinister, *OD* oculus dextrus, *VA1* Visual acuity before treatment, *VA2* Visual acuity after treatment, *HM* Hand motion, *VRC* Voriconazole; *ITR*: Itraconazole, *AMB* Amphotricin B, *Eviscer* Evisceration, *PKP* Penetrating Keratoplasty

#### Patient 1

**Fig. 1:**
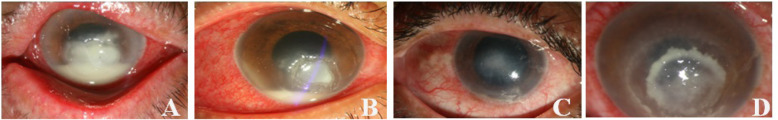
Slit-lamp photographs of patients. Hypopyon can be seen in the anterior chamber of patient 1 (**A**) and patient 2 (**B**), A grayish, superficial stromal infiltrate with indistinct margins can be seen in patient 3 (**C**) patient 4 (**D**)

**Fig. 2:**
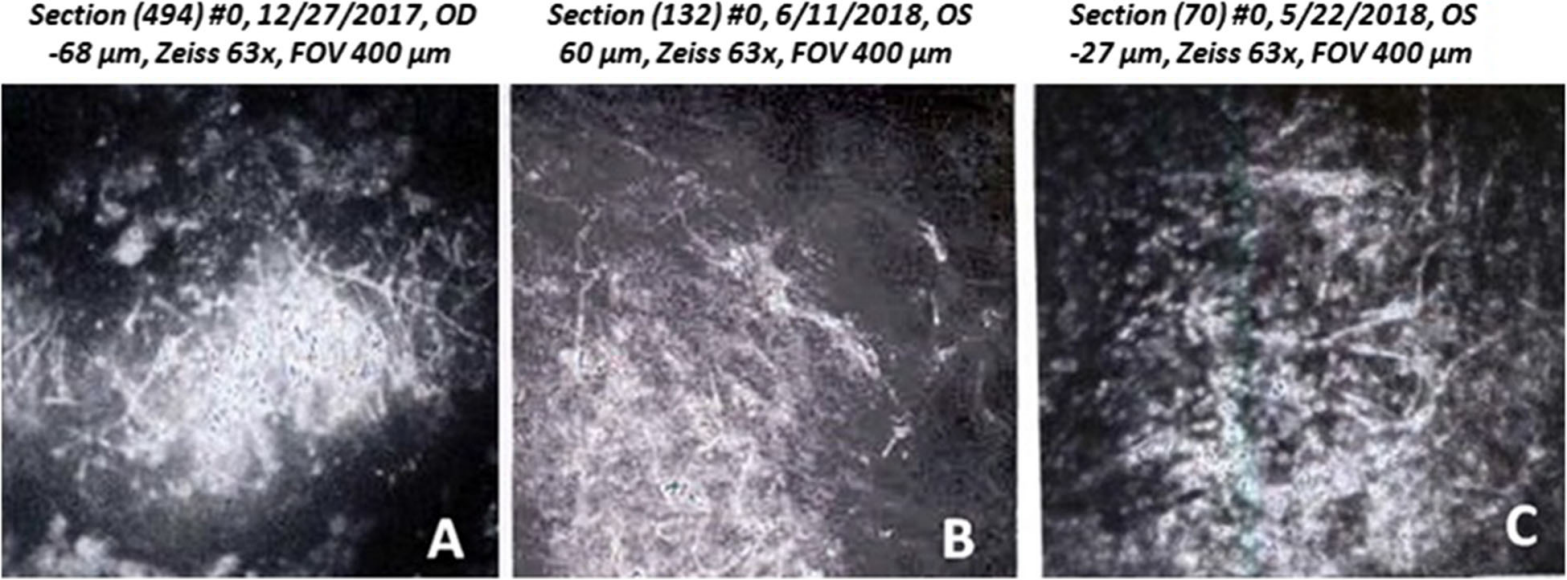
In vivo confocal microscopy (IVCM) from patient 1 (**A**), patient 2 (**B**) and patient 3 (**C**) showing a mass of interlocking and diffuse, hyper-reflective and branching white lines indicating filamentus fungi

The patient was a 46-year-old woman who felt dust particles blow into her right eye during farm work. On clinical examination, her best-corrected visual acuity (BCVA) was counting fingers (CF) from 50 cm. On slit-lamp examination she had a grayish, superficial stromal infiltration with epithelial defect and indistinct margins. Hypopyon was detected in the anterior chamber (Fig. 1A). Examination of the cornea with the IVCM showed a mass of interlocking and branching white lines in the area of the infiltrate, this feature suggesting filamentous fungus keratitis (Fig. 2A). Based on clinical picture and the result of corneal scraping treatment were begun with oral itraconazole (200 mg/day), topical 1% voriconazole hourly, topical 0.1% amphotricin B 2 time/day and topical 0.5% levofloxacin 4 times/day. After medications the hypopyon resolved in a few days, the focal infiltrate resolved within 2 weeks and the BCVA in her right eye improved to 20/160 within 2 months.

#### Patient 2

A 69-year-old man attended our hospital with a sudden loss of visual acuity in left eye after trauma with plant material. On clinical examination, her BCVA was CF from 50 cm. Slit-lamp examination showed corneal ulcer and associated hypopyon (Fig. 1B). Examination of the cornea with the IVCM showed branching white lines in the area of the infiltrate, this feature suggesting filamentous fungus keratitis (Fig. 2B). Based on clinical picture and the result of corneal scraping the patient was treated with application topical 1% voriconazole hourly and levofloxacin (4 times/day) for 4 weeks. Two weeks later hypopyon and focal infiltrate resolved. The BCVA in her left eye improved to 20/200 within 1 month.

#### Patient 3

The patient was a 30-year-old man whose left eye was injured by a wood particle during woodworking. He had history of pain, redness, sensation of foreign body, excessive tearing and diminution of vision in the left eye. On initial examination, her BCVA was CF from 50 cm. On slit-lamp examination he had grayish white lesion involving the corneal endodermis, pyoderma in the endothelium, and peripheral corneal edema. Hypopyon was detected in the anterior chamber (Fig. 1C). Examination of the cornea with the IVCM showed branching white lines in the area of the infiltrate, this feature suggesting filamentous fungus keratitis (Fig. 2C). Based on clinical picture and the result of corneal scraping treatment were begun with topical 1% voriconazole hourly. After medications the hypopyon resolved in a few days, the focal infiltrate resolved within 2 weeks and the BCVA in her left eye improved to 20/19 within 2 months.

#### Patient 4

A 37-year-old man whose right eye was injured by a branch of a tree during farm work visited ophthalmology outpatient unit at our tertiary referral center. He had progressive pain, sensation of foreign body, excessive tearing and redness in the right eye. On initial examination, the BCVA of the right eye was found to be 20/60. Slit lamp examination of the affected eye revealed superficial corneal stromal infiltrate with gray-white in color and feathery edged margins. Satellite lesions and hypopyon were not seen. Based on clinical picture and the result of corneal scraping treatment were begun with topical 1% voriconazole hourly. After medications the focal infiltrate resolved within 10 days and the BCVA in her light eye improved to 20/16 within 1 month.

### Mycology characteristics

Direct microscopic examination of scrapes using KOH and gram staining reveals septate hyphae. Gray-white fungal colonies grew on sabouraud dextrose agar after 3 days’ incubation at 30 °C. Identification was subsequently made from microscopic examination of the colonies that showed ovoid conidia with hyphae which are features characteristic of *P. boydii.* Using NCBI BLAST, the fungi isolates were identified as *P. boydii* with 100% identity in the GenBank database.

The Table 2 shows the MICs of various antifungal drugs against *P. boydii* isolates. The *P. boydii* isolates were sensitive to voriconazole, itraconazole, posaconazole, caspofungin and Chlor hexidin, while resistant to amphotericin B, 5-flucytosine, fluconazole and natamycin.

**Table 2:** MICs (μg/ml) of different antifungal drugs on the *P. boydii* isolates

	AMB	5FC	FLU	ITR	VRC	PSO	MICO	CAS	Chlorhex	Nata
**Patient 1**	4	≥ 64	64	1	0.5	1	0.5	2	0.008	2
**Patient 2**	4	≥ 64	≥ 64	1	0.5	0.5	0.5	0.5	0.008	2
**Patient 3**	4	≥ 64	≥ 64	0.5	0.25	1	0.5	0.25	0.008	4
**Patient 4**	4	≥ 64	64	0.5	0.5	1	0.5	0.5	0.008	2

Abbreviations: *AMB* Amphotricin B, *FC* 5 flucytosine, *FLU* Fluconazole, *ITR* Itraconazole, *VRC* Voriconazole, *PSO* Posaconazole, *MICO* Miconazole, *CAS* Caspofungin, *Chlorhex* Chlorhexidin, *Nata* Natamycin

## Discussion

*P. boydii* and its asexual form (*S. apiospermum*) belong to the group of septate filamentous fungi are uncommon cause of mycotic keratitis in humans. A review of the literature showed that *P. boydii* is the most common species of *Scedosporium boydii* complex (*S. aurantiacum*, *P. minutispora*, *S. dehoogii*, *S. dehogii*, and *P. boydii*) that reported as Pseudallescheria keratitis [12, 13]. To the best of our knowledge, fungal keratitis caused by *P. boydii* has only been reported in the literature as single cases [14–17]. For the first time, we describe the clinical and mycological features of four fungal keratitis caused by *P. boydii* over a one-year period in a training hospital. Rathi et al. reported that the principal risk factor for Pseudallescheria keratitis was ocular trauma [8]. The Pseudallescheria species are commonly found in soil, water, and sewage [9]. Thus, the principal risk factor for Pseudallescheria keratitis is ocular trauma and contamination by concomitant soil contamination [7, 9]. All of our patients (3 patient injured by plant material and 1 by dust particles) also gave a similar history of trauma.

Treatment of fungal keratitis remains a difficult problem for each ophthalmologist because there is no ideal antimycotic drug [12]. A delayed diagnosis and the use of conventional antifungals often lead to poor prognosis in Pseudallescheria keratitis [6]. Treatment is often based on clinical response [7]. Voriconazole was reported to be effective against *S. apiospermum* [7, 18]. However, it seems to be less effective against another *Scedosporium* species [19]. Viconazole treatment failure has sometimes resulted in the surgical procedure such as penetrating keratoplasty (PKP) [20]. Surgical procedure can result in complications such as pain, postoperative inflammation and graft rejection [21]. Hernandez et al. and Nulens et al. have reported voriconazole to be effective against fungal keratitis caused by *S. apiospermum* [22, 23]. Ramakrishnan and colleagues reported in their series, natamycin and fluconazole combination therapy succeeded in seven of 10 cases of ocular infections caused by *S. apiospermum* [13]. Patients in our study were prescribed voriconzole alone without needing for surgical interventions and PKP. All patients showed complete healing of their ulcers with this monotherapy. In our antifungal susceptibility tests, all four strains displayed in vitro resistance to amphotericin B and fluconazole and susceptible to voriconazole and itraconazole. In vitro sensitivity tests for caspofungin displayed three isolates resistance and one isolate susceptible to this antifungal agent. There is few investigation of the antifungal susceptibility against *P. boydi* isolated from keratitis. Sicilia and colleagues have reported the isolated strain displayed in vitro resistance to all the antifungal agents (amphotericin B, fluconazole, itraconazole, ketoconazole and 5 -fluorocytosine) tested [7]. These results are some similar to our results. The chlorhexidine MICs of all isolates of *P. boydi* tested were 0.008 μg/ml. Four patients  with Fusarium keratitis were successfully treated with chlorhexidine 0.02% eye drops by Oliveira dos Santos et al. [24]. The efficacy of chlorhexidine for treating fungal keratitis has been confirmed by studies [24, 25]. There is a need for further research on the use of chlorhexidine in Pseudallescheria keratitis.

In conclusion, these patients bold the importance of determining causative organism of fungal keratitis and their antibiotic susceptibility. Culture findings are limited in identifying organisms. Sequencing of polymerase chain reaction-amplified DNA is good for accurate and rapid identification of species that can be helpful for optimize treatment.

## Data Availability

Not applicable.

## Abbreviations

ITS: Internal transcribed spacer region
CLSI: Clinical and Laboratory Standards Institute
IVCM: In vivo confocal microscopy
KOH: Potassium Hydroxide
BLAST: Basic Local Alignment Search Tool
MICs: Minimum inhibitory concentration
BCVA: Best-corrected visual acuity
CF: Counting fingers
